# Systems Biology of Microbial Exopolysaccharides Production

**DOI:** 10.3389/fbioe.2015.00200

**Published:** 2015-12-18

**Authors:** Ozlem Ates

**Affiliations:** ^1^Department of Medical Services and Techniques, Nisantasi University, Istanbul, Turkey

**Keywords:** EPS, microbial production, exopolysaccharides, systems biology, xanthan, levan, pullulan, dextran

## Abstract

Exopolysaccharides (EPSs) produced by diverse group of microbial systems are rapidly emerging as new and industrially important biomaterials. Due to their unique and complex chemical structures and many interesting physicochemical and rheological properties with novel functionality, the microbial EPSs find wide range of commercial applications in various fields of the economy such as food, feed, packaging, chemical, textile, cosmetics and pharmaceutical industry, agriculture, and medicine. EPSs are mainly associated with high-value applications, and they have received considerable research attention over recent decades with their biocompatibility, biodegradability, and both environmental and human compatibility. However, only a few microbial EPSs have achieved to be used commercially due to their high production costs. The emerging need to overcome economic hurdles and the increasing significance of microbial EPSs in industrial and medical biotechnology call for the elucidation of the interrelations between metabolic pathways and EPS biosynthesis mechanism in order to control and hence enhance its microbial productivity. Moreover, a better understanding of biosynthesis mechanism is a significant issue for improvement of product quality and properties and also for the design of novel strains. Therefore, a systems-based approach constitutes an important step toward understanding the interplay between metabolism and EPS biosynthesis and further enhances its metabolic performance for industrial application. In this review, primarily the microbial EPSs, their biosynthesis mechanism, and important factors for their production will be discussed. After this brief introduction, recent literature on the application of omics technologies and systems biology tools for the improvement of production yields will be critically evaluated. Special focus will be given to EPSs with high market value such as xanthan, levan, pullulan, and dextran.

## Introduction

Biopolymer (also called renewable polymers) is used as a term to describe polymers produced by biological systems and polymers that are not synthesized chemically but are derived from biological starting materials such as amino acids, sugars, and natural fats (Tang et al., 2012). Consequently, biopolymers can be classified as synthetic or natural polymers (Vroman and Tighzert, 2009). The biopolymers are superior to petrochemical-derived polymers in several aspects that include biocompatibility, biodegradability, and both environmental and human compatibility. Although petroleum-based polymers have negative effects to environment and humanity such as toxicity, defiance to biodegradation, and waste accumulation, they have been used in a variety of industrial applications. Therefore, in response to these problems, biopolymers are a suitable alternative that the researchers were looking (Keshavarz and Roy, 2010).

Today, several microorganisms are identified as microbial biopolymer producers and these polymers can be found as attached to the cell surface or extracted from the fermentation medium. Bacteria use these microbial biopolymers as storage materials in response to particular environmental stresses (Sanchez-Garcia et al., 2010). Due to their biological functions microbial polysaccharides can be generally classified as intracellular storage polysaccharides (glycogen), capsular polysaccharides (e.g., K30 O-Antigen), and extracellular bacterial polysaccharides (for example, levan, xanthan, sphingan, alginate, pullulan, cellulose, etc.), which are important for biofilm formation and pathogenicity (Schmid and Sieber, 2015).

Microbial polysaccharides that are produced by microorganisms and secreted out of the cell are defined as exopolysaccharides (EPSs). In nature, they have a significant role for protection of the cell, adhesion of bacteria tosolid surfaces, and participating in cell-to-cell interactions (Nicolaus et al., 2010). In recent years, there is a significant interest on microbial EPSs since they have different structural and functional properties (Morris and Harding, 2009). EPSs are important resources for hydrocolloids used in food, pharmaceutical, chemical, and many other industries (Ahmad et al., 2015). Due to their many interesting physicochemical and rheological properties with novel functionality, the microbial EPSs act as new biomaterials and find a wide range of applications in many industrial sectors such as textiles, detergents, adhesives, microbial enhanced oil recovery (MEOR), wastewater treatment, dredging, brewing, downstream processing, cosmetology, pharmacology, and food additives (Rühmann et al., 2015). Xanthan, dextran, and pullulan are examples of microbial polysaccharides with a considerable market due to their exceptional properties. However, plant and algal polysaccharides such as starch, galactomannans, pectin, carrageenan, and alginate still include a major part of the hydrocolloid market, which has a market value of 4 million US dollars in 2008, 3.9 billion US dollars in 2012, and this value is expected to reach 7 billion US dollars by 2019 (Williams et al., 2007; Patel and Prajapati, 2013). Since microbial EPSs enable fast and high yielding production processes under controlled conditions, they are economically competitive to the plant and algal origin polysaccharides, which are affected by climatological and geological environmental conditions (Kaur et al., 2014). Although there is an increased attraction for microbial EPSs in industrial and medical applications and they are related with high-value applications, only a few bacterial EPSs have achieved to be used commercially such as xanthan, gellan, and dextran due to high production costs (Freitas et al., 2011; Llamas et al., 2012).

Due to the exceptionally high production costs, microbial EPSs could never find their proper place in the polymer market and therefore, high-level EPSs producing microbial systems gain escalating industrial importance. Increasing significance of EPSs in industrial and medical biotechnology calls for the elucidation of the interrelations between metabolic pathways and biosynthesis mechanism in order to control and hence improve microbial productivity. Therefore, extensive interest has been dedicated to understand bacterial EPSs biosynthesis mechanism and pathways and enhance productivity within the past years. Omics technologies such as genome sequencing, functional genomics, protein structure analysis, and new bioinformatics tools have been used to identify new EPS biosynthesis pathways and understand the principles of EPS formation (Schmid and Sieber, 2015).

**Figure 1:**
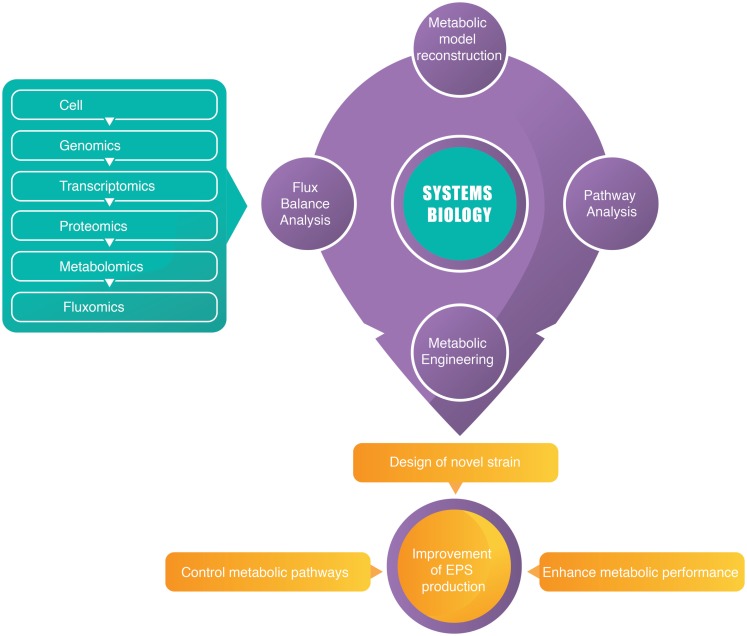
**A schematic diagram of integration of omics studies with systems biology**.

A modeling approach that linked omics data and the simulation of variable expression and enzyme activity will provide information about a cell’s macromolecular machinery (Lerman et al., 2012). For this purpose, genome-based and genome-scale metabolic reconstructions can be used to understand and predict phenotypes of a microbial species (Hanemaaijer et al., 2015). Therefore, a systems biology approach constitutes an important step toward understanding the interplay between metabolism and microbial EPS biosynthesis and further enhances its metabolic performance for industrial application. Figure 1 demonstrated a brief summary of integration of omics studies with systems biology.

In this review after a brief description of the microbial EPSs, biosynthesis mechanism and important factors for their production, recent literature on the application of omics technologies and systems biology tools for the improvement of production yields will be critically evaluated. Microbial EPSs with high market value such as xanthan, dextran, scleroglucan, pullulan, and levan are specially focused.

## Microbial Exopolysaccharides

The biopolymers produced by microorganisms were categorized to four main groups: polyesters, polyamides, inorganic polyanhydrides, and polysaccharides (Crescenzi and Dentini, 1996; Rehm, 2009, 2010). Since the microbial biopolymers serve as reserve material or as part of a protective mechanism, the biopolymer producer microorganisms have significant advantages under certain environmental conditions (Rehm, 2010). The first bacterial polymer dextran was discovered by Pasteur (1861) in the mid-nineteenth century as a microbial product in wine and the bacterium *Leuconostoc mesenteriodes* was identified by Van Tieghem (1878) as dextran producer strain.

The bacterial polysaccharides that are synthesized and secreted by various microorganisms into the extracellular environment either as soluble or insoluble polymers are defined as EPSs. Their compositions, functions, chemical, and physical properties that establish their primary conformation vary from one bacterial species to another. EPSs are composed of mainly of carbohydrates (a wide range of sugar residues) and some non-carbohydrate substituents (such as acetate, pyruvate, succinate, and phosphate) (Vu et al., 2009; Nicolaus et al., 2010; Llamas et al., 2012; Staudt et al., 2012).

Most of EPSs producer bacteria have been described to produce either homo or heteropolysaccharide (Kumar et al., 2007). On the other hand, bacteria (*Serratia marcescens*, *Aeromonas salominicida*, and *Pseudomonas* sp. strain NCIB 2021) that were able to produce two different polysaccharides have been reported (Kwon et al., 1994). Due to linkage bonds and nature of monomeric units, homopolysaccharides can be categorized as α-d-glucans, β-d-glucans, fructans, and polygalactan. d-glucose, d-galactose, l-rhamnose, and *N*-acetylglucosamine (GlcNAc), *N*-acetylgalactosamine (GalNAc), or glucuronic acid (GlcA) are the repeating units of heteropolysaccharides and occasionally non-carbohydrate substituent such as phosphate, acetyl, and glycerol. Homopolysaccharides and heteropolysaccharides are also dissimilar in synthetic enzymes and site of synthesis. Biosynthesis of homopolysaccharides requires specific substrates like sucrose, while the residues of heteropolysaccharide are produced intracellularly and precursors are located across the membrane by isoprenoid glycosyl carrier lipids for extracellular polymerization (Nwodo et al., 2012). EPSs have also been classified in seven categories based on their functionality by Flemming and Wingender (2010) as constructive or structural (serve in the matrix help water retention and cell protection), sorptive (composed of charged polymers), surface-active (including molecules with amphiphilic behavior), active, informative, redox-active, and nutritive.

EPS affords self-protection for cells from desiccation, predation, the effects of antibiotics, antimicrobial substances, antibodies, bacteriophages and adherence to other bacteria, animal, and plant tissues under different stress conditions such as biotic stress, competition, and abiotic stresses, including temperature, light intensity, or pH (Mata et al., 2006; Kumar et al., 2007; Kumar and Mody, 2009; Ordax et al., 2010; Donot et al., 2012; Staudt et al., 2012). Additionally, EPS supplies bacterial aggregation, surface attachment, and symbiosis of plant-microbe; hence, it is a crucial property for wastewater treatment and soil aggregation. Furthermore, pathogenicity of a microorganism is related with the production of capsular EPS and depends on the rate and amount of EPS synthesis (Kumar et al., 2007).

Microorganisms are often linked with a high cellular density biofilm and its stability is controlled by EPSs through interactions between the polysaccharide chains. Moreover, microbial diversity is biologically supported by EPS to constitute a substrate for the microbial growth. Biofilm formation plays a crucial role both in adhesion and in adaptation of bacteria to the physicochemical conditions of the environment (Donot et al., 2012).

Environmental factors and specific culture conditions such as pH, temperature, carbon-to-nitrogen (C/N) ratio, oxygenation rate, and carbon sources can impact EPS production. EPS composition can be altered by conditional changes (differing monosaccharides or by monosaccharide molar ratio). For instance, due to the carbon source, *Lactobacillus casei* has been shown to alter the chemical composition of its EPS (Staudt et al., 2012). Furthermore, production of microbial EPSs in bioreactors enables to optimize growth and production yields by studying physiology and genetic engineering (Delbarre-Ladrat et al., 2014).

Due to their unique and complex chemical structures that offers beneficial bioactive functions, biocompatibility, and biodegradability, microbial EPSs have find a wide range of application areas in chemical, food, pharmaceutical, cosmetics, packaging industries, agriculture, and medicine in which they can be used as adhesives, absorbents, lubricants, soil conditioners, cosmetic, drug delivery vehicles, textiles, high-strength materials, emulsifiers, viscosifiers, suspending, and chelating agents. In recent years, several novel bacterial EPSs have been isolated and identified; however, a few of them have achieved to have significant commercial value due to the high production costs (Mata et al., 2006; Kumar et al., 2007; Nicolaus et al., 2010; Freitas et al., 2011; Llamas et al., 2012; Delbarre-Ladrat et al., 2014). Bacterial EPSs such as xanthan, gellan, dextran, and curdlan with superior physical and chemical properties are used instead of plant (guar gum or pectin) or algae (e.g., carrageenan or alginate) polysaccharides in traditional applications (Kumar and Mody, 2009; Nicolaus et al., 2010; Freitas et al., 2011; Liang and Wang, 2015). Other bacterial EPSs such as levan, pullulan, and wellan with unique properties and biological activities have found new commercial opportunities (Freitas et al., 2011).

GalactoPol, which is synthesized by *Pseudomonas oleovorans* and composed mainly of galactose, and a fucose containing EPS FucoPol that is synthesized by *Enterobacter* A47 have been recently reported bacterial EPSs with great commercial potential. Almost 30 species of lactic acid bacteria (LAB) are also known as polysaccharide producers and one of the commercial EPS dextran producer *Leuconostoc mesenteroides* is a LAB; however, low production yields avoid LAB species to be exploited commercially. Besides, *lactobacilli* are GRAS (generally recognized as safe) bacteria and their EPS could be utilized in foods (Badel et al., 2011).

After the discovery of the various EPSs, the activities of enzymes related with EPS production were investigated and radioisotope-labeled precursors were used to elucidate the metabolic pathways for microbial biosynthesis. Moreover, understanding the molecular and regulatory mechanisms behind the biosynthesis of microbial polymers is an essential requirement for engineering bacteria leading to production of tailor-made biopolymers with high-value applications for industrial and medical applications with an economic cost (Rehm, 2009, 2010).

## Bacterial Synthesis of Exopolysaccharides

Extensive progress has been made in elucidating the biosynthetic and genetic mechanisms of biosynthesis of bacterial polysaccharides in recent years. The mechanism of biosynthesis and the precursors required illustrate diversity for different classes of EPSs. EPSs are synthesized by bacteria extracellularly or intracellularly (Boels et al., 2001; Kumar et al., 2007; Badel et al., 2011; Freitas et al., 2011; Li and Wang, 2012). Genes required for EPS production are responsible for encoding regulation, chain-length determination, repeat-unit assembly, polymerization, and export. The mechanism regulating EPS biosynthesis is a challenged topic to be understood despite accumulating knowledge of EPS gene organization (Péant et al., 2005). Regulation of EPS biosynthesis is related with various physiological and metabolic parameters such as the availability of sugar precursors and the expression level of enzymes (Delbarre-Ladrat et al., 2014). Information on genetics of certain EPS like xanthan is abundant; however, genetic data for other EPS synthesis (i.e., pullulan) is still limited (Donot et al., 2012).

Bacterial EPSs are mostly generated intracellularly and exported to the extracellular environment with the exception of homopolysaccharides such as dextran, levan, and mutan that are synthesized outside the cells by the action of secreted enzymes that convert the substrate into the polymer. The enzymes involved in EPS synthesis are found at different regions of the cell and can be characterized into four categories. The first group is intracellular enzymes such as hexokinase, which phosphorylates glucose (Glc) to glucose-6-phosphate (Glc-6-P). They are also involved in other cellular metabolisms. The second group is required to catalyze conversion of sugar nucleotides. Uridine-5′-diphosphate (UDP)-glucose pyrophosphorylase that catalyzes the conversion of Glc-1-P to UDP-Glc, which is one of the key molecules in EPS synthesis can be given as an example for this class of enzymes. Another enzyme group is glycosyltransferases (GTFs) that are located in the cell periplasmic membrane. The sugar nucleotides are transferred by GTFs to a repeating unit attached to glycosyl carrier lipid. The enzymatic functions, the structures, and identification of the genes that encode GTFs has been investigated intense and due to amino acid sequence similarities more than 94 GTF families were reported in the Carbohydrate-Active EnZymes (CAZy) database (http://www.cazy.org) (Li and Wang, 2012). The last class is presumably involved in the polymerization of the macromolecules and situated outside the cell membrane and the cell wall (Kumar et al., 2007).

**Figure 2:**
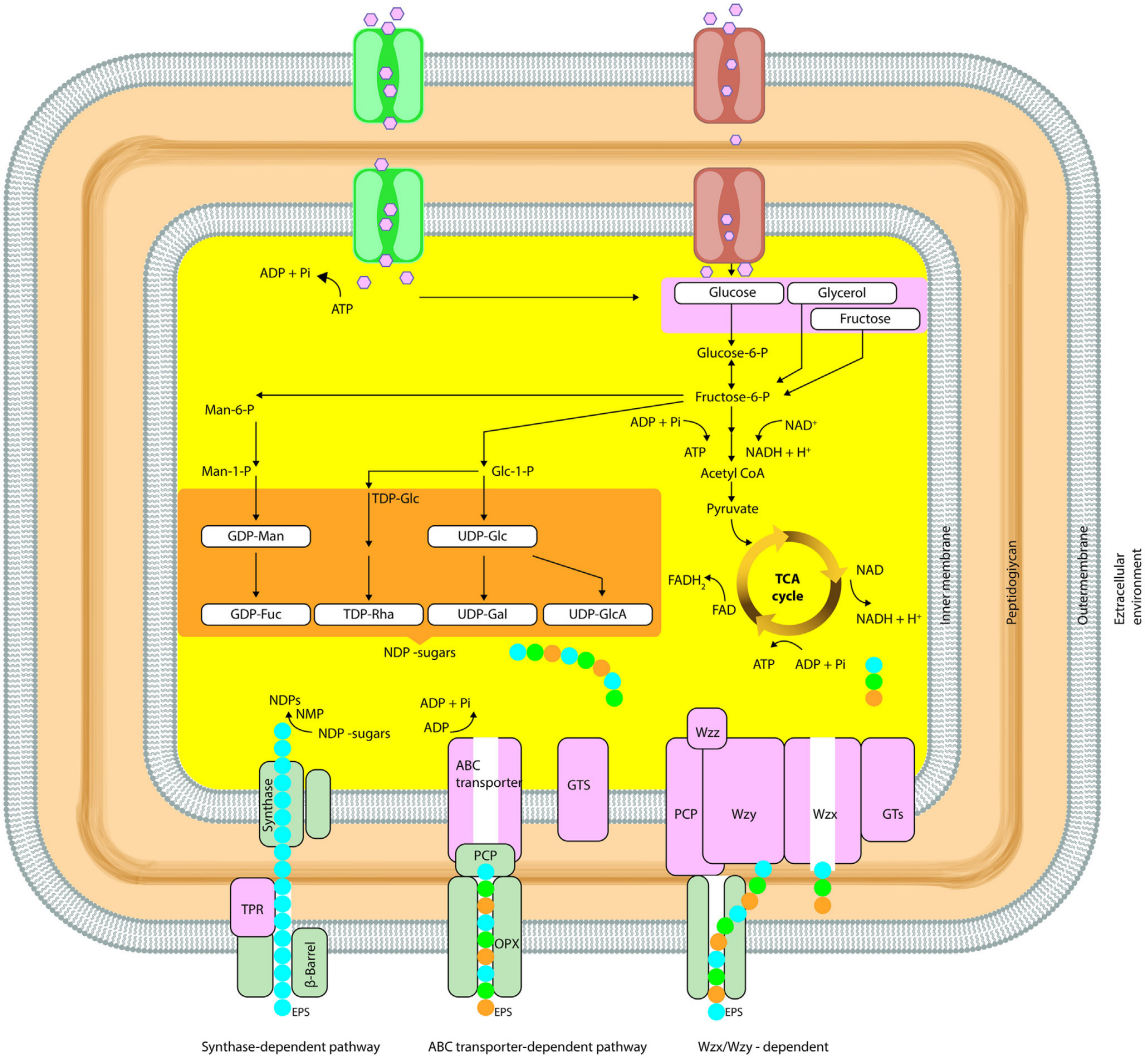
**Simplified schematic diagram summarizing the biosynthetic pathways (Synthase dependent, ABC transporter, Wzx/Wzy dependent) involved in microbial EPS synthesis (OPX, outer-membrane polysaccharide; PCP, polysaccharide copolymerase; TPR, tetratricopeptide repeat proteins)**.

The general mechanisms for the production of bacterial polysaccharides are Wzx/Wzy-dependent pathway, the ATP-binding cassette (ABC) transporter-dependent pathway, the synthase-dependent pathway, and the extracellular synthesis by use of a single sucrase protein. Inside the cell, the precursor molecules are transformed by enzymes and produce activated sugars/sugar acids in the first three mechanisms. Alternatively, in extracellular production pathway by direct addition of monosaccharides obtained by cleavage of di- or trisaccharides, the polymer strand is elongated (Schmid and Sieber, 2015).These general mechanisms of EPS biosynthesis were demonstrated in detail in Figure 2.

In the Wzx/Wzy-dependent process, activated sugars are linked in a specific sequence to a lipid carrier by GTFs until the repeating unit is formed involving a Wzy protein. In the Wzx/Wzy-independent (ABC transporter-dependent) pathway, polymerization occurs at the cytoplasmic side of the inner membrane. The genes, which are required for high-level polymerization and surface assembly, are described as wza (encoding an outer-membrane protein), wzb (encoding an acid phosphatase), and wzc (encoding an inner-membrane tyrosine autokinase). In most Gram-negative bacteria (i.e., *Erwinia* spp., *Methylobacillus* sp. strain 12S, *Rhizobium* spp., and *Xanthomonas campestris*), EPS biosynthesis and export have been reported to occur via the Wzx/Wzy-independent and Wzx/Wzy-dependent pathway (Arco et al., 2005; Cescutti et al., 2010; Freitas et al., 2011).

EPS secretion can occur in the presence or absence of a lipid acceptor molecule in the synthase-dependent pathway, which secretes complete polymer strands across the membranes and the cell wall, and is not dependent of a flippase for translocating repeat units. In this system, the polymerization and the translocation process are performed simultaneously by a single synthase protein, a membrane-embedded glycosyl transferase. These pathways are often utilized for the assembly of homopolymers requiring only one type of sugar precursor such as curdlan [β-(1-3)-linked glucose monomers] or bacterial cellulose [β-(1-4)-linked glucose units] (Rehm, 2010; Whitney and Howell, 2013; Schmid and Sieber, 2015). Regulation of polymerization is implemented by an inner-membrane receptor, in Gram-negative synthase-dependent secretion systems such as *P. aeruginosa* alginate and *Gluconacetobacter xylinus* cellulose (Whitney and Howell, 2013).

In extracellular synthesis, polymerization reaction occurs as transfer of a monosaccharide from a disaccharide to a growing polysaccharide chain in the extracellular environment. This type of production of EPSs is uncomplicated; independent of the central carbon metabolism besides there is a limited variation in structure. The extracellular EPS synthesis can occur for homopolysaccharides (dextran, levan, and mutan) by extracellular GTF (Boels et al., 2001; Finore et al., 2014).

The intracellular biosynthesis of homo- and heteropolysaccharides includes production of (ir)regular repeating units from sugar nucleotide precursors, which are also involved in the biosynthesis of several cell wall components and can therefore be considered essential for growth (Boels et al., 2001; Nicolaus et al., 2010; Li and Wang, 2012). Direct precursors for bacterial EPS biosynthesis are formed intracellulary from intermediates of the central carbon metabolism. The precursors and donor monomers for the biosynthesis of most repeating units are sugar nucleotides such as nucleoside diphosphate sugars (such as ADP-glucose), nucleoside diphosphate sugar acids (such as GDP-mannuronic acid), and nucleoside diphosphate sugar derivatives [such as UDP-glucose, UDP-*N*-acetyl glucosamine, UDP-galactose, and deoxythymidine diphosphate (dTDP)-rhamnose] (Barreto et al., 2005; Péant et al., 2005; Rehm, 2010). These sugars can be transported by basically three different ways: ATP hydrolysis coupled to sugar translocation via a sugar transport ATPase, the import with coupled to transport of ions and other solutes, and transport via the phosphoenolpyruvate (PEP) transport system (PTS) (Barreto et al., 2005; Péant et al., 2005).

GTFs (EC 2.4.x.y) catalyze heteropolysaccharide biosynthesis which has numerous intracellular steps and only the last step that polymerization of repeating units occurs is extracellular. Depending on substrate type, uptake of sugars is achieved through a passive or an active transport system by the cell in the first step. Subsequently, the substrate is catabolized in the cytoplasm through glycolysis and sugar nucleotides are formed. The biosynthesis of activated precursors [energy-rich monosaccharides, mainly nucleoside diphosphate sugars (NDP-sugars)], which are derived from phosphorylated sugars is occurred. Finally, EPS is secreted to extracellular environment therefore their secretion from cytoplasm through cell membrane without compromising the critical barrier properties is a challenging process (Badel et al., 2011; Freitas et al., 2011).

Conversely, homopolysaccharides are synthesized extracellularly by GTFs. This class is defined as glycansucrases class (E.C. 2.4.x.y) and dissimilar to classical Leloir-type GTF they utilize sucrose as donor substrate instead of nucleotide-sugars. The transfer of monosaccharides, generating a glycosidic bond, from activated molecules to an acceptor molecule is catalyzed by these enzymes. Energy released by degredation of sugars is used to catalyze transfer of a glycosyl residue on forming polysaccharide. Due to the product of biosynthesis, the enzymes can be differentiated between transglucosydases (EC 2.4.1.y) and transfructosydases (EC 2.4.1.y or 2.y). Transglucosidases class includes dextransucrase, mutansucrase, and reuteransucrase (EC 2.4.1.5), which are high molecular weight extracellular enzymes and catalyze hydrolysis of sucrose to glucose and fructose and glucosyl transfer on carbohydrate or non-carbohydrate compounds. EPS structures can be varied based on different enzymes intervention and the synthesis of each polysaccharide is catalyzed by a specific GTF; therefore, two products encoded by two genes of *gtf* result in two different EPS. In addition, the enzyme conformation affects branching degree of homopolysaccharides. Levansucrases (EC 2.4.1.10) and inulosucrases (EC 2.4.1.9) from transfructosidases class produce levan and inulin type fructans. *Ftf* genes are induced under stress conditions and sucrose hydrolysis and fructosyl transfer on fructan polymerized chain or syntheses of tri- or tetrasaccharides are catalyzed. In fructan, glucose is the non-terminal reducing residue (G-Fn) (Badel et al., 2011).

## Omics Studies and Systems Biology and of Microbial Biopolymer and Microbial EPS Production

Systems biology offers valuable application areas in molecular sciences, medicine, pharmacy, and engineering such as pathway-based biomarkers and diagnosis, systematic measurement and modeling of genetic interactions, systems biology of stem cells, identification of disease genes, drug design, strain development, bioprocess optimization (Medina-Cleghorn and Nomura, 2013).

Strain improvement using systems level analysis of metabolic, gene regulatory and signaling networks, and integration of omics data are the most focused subjects of systems biology. Biochemical and bioprocess engineering principles are applied for optimization of upstream-to-downstream bioprocesses at first stages of strain development (Barrett et al., 2006). Process development has been a supporter of the scientific achievements in systems biology, mostly in the areas of transcriptomics, proteomics, metabolomics, and fluxomics with availability of genome sequences for production organisms. The applications of systems biology in industry become a challenged subject (Otero and Nielsen, 2010).

In recent years, the enormous amounts of genome sequencing projects have resulted in accumulation of complete genome sequence information for a number of species. This information is valuable for understanding biological capabilities of organisms and biological processes such as signal transduction and cellular metabolism at the system levels. Therefore, an ever-increasing number of models for bacteria and more papers that describe new reconstruction tools and improvements have been published. Genome-scale constraint-based metabolic models have been reconstructed for several organisms and such constraint-based models can be quickly generated by software packages using an organism’s genomic, biochemical, and physiological data. These metabolic models have been used to integrate high-throughput data understand cellular metabolism, to develop metabolic engineering strategies, to design media and processes, to consider theoretical capabilities, and to control the process online, which illustrates its usefulness for development and optimization of process. Metabolic models are used for generating new hypotheses and targeting promising areas in engineering field. Metabolic engineering studies have been performed to modify microbes to produce industrially relevant biochemicals and biofuels such as ethanol and higher alcohols, fatty acids, amino acids, shikimate precursors, terpenoids, polyketides, and polymer precursors (e.g., 1,4-butanediol) (Henry et al., 2010; Baart and Martens, 2012; Xu et al., 2013; Long et al., 2015; Simeonidis and Price, 2015).

**Figure 3:**
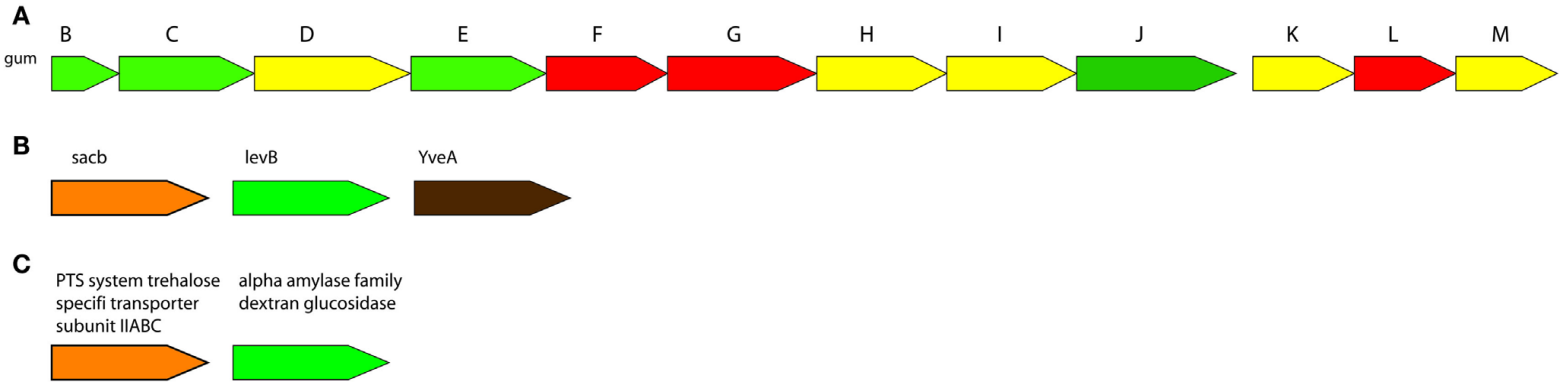
**Gene clusters of significantly important microbial EPSs biosynthesis**. **(A)**: Xanthan biosynthesis by *Xanthomonas campestris* pv. *campestris* ATCC 33913, **(B)**: Levan biosynthesis by *Bacillus subtilis* subsp. *subtilis* 6051-HGW (bsh), **(C)**: Dextran biosynthesis by *Streptococcus intermedius* JTH08 (sie). Conserved operan informations were obtained from ODB Operon Database (Okuda and Yoshizawa, 2011).

Genome-scale metabolic networks have great achievement in development of metabolic engineering strategies for strain improvement mainly in five industrial fields: food and nutrients, biopharmaceuticals, biopolymer materials, microbial biofuels, and bioremediation. Metabolic models are built to improve the yield of fermentation by products and explore metabolic mechanisms and processes in food and nutrients industry. Several biopharmaceuticals and the productivity of useful biopolymers and their precursors have improved by genome-scale metabolic model-guided metabolic engineering strategy (Xu et al., 2013). Dupont’s near-decade long optimization of *Escherichia coli* for bioproduction of 1,3-propanediol is an important genome-scale metabolic engineering application (Nakamura and Whited, 2003). The industrially optimized strain required up to 26 genomic changes including insertions, deletions, and regulatory modifications. Recent advances in constraint-based modeling have enabled *in silico* prediction of genomic targets for the enhancement of strain performance or product yield (Esvelt and Wang, 2013). The engineering strategies have been successfully implemented for the improvements in the yield or production process, alterations in the degree of polymerization, removal of side chains or non-sugar substituents, or heterologous expression of EPS biosynthesis gene clusters (Becker, 2015). Additionally, the gene clusters of significant EPSs were figured out in Figure 3.

Due to their superior properties, wide application areas, there is a high demand to improve microbial EPS production with an economical cost. Therefore, the omics data and tools were utilized to perform systems biology approaches to understand and control EPS biosynthesis mechanism, design novel strains, and enhance productivity.

Natural or engineered microorganisms could synthesize many biopolymers and their monomers such as poly-3-hydroxyalkanates (PHAs), polylactic acid (PLA), polysaccaharides, carboxylic acids, and butanediols. Systems biology approach and genome-scale metabolic models-guided metabolic engineering strategies have been successfully employed to enhance the productivity of useful biopolymers and their precursors (Xu et al., 2013).

Jung et al. reported direct synthesis of PLA, which is a promising biomass-derived homopolymer and its copolymer, poly (3-hydroxybutyrateco- lactate), P (3HB-co-LA), by direct fermentation of metabolically engineered *E. coli*. In typical conditions, PLA production involves two steps fermentative production of lactic acid followed by chemical polymerization with low production yields. In this study, *in silico* genome-scale metabolic flux analysis was performed to determine metabolic engineering targets to improve *E. coli* strain. The engineering process was achieved by knocking out the ackA, ppc, and adhE genes and by replacing the promoters of the ldhA and acs genes with the trc promoter, and therefore, an 11 wt% enhancement of PLA production was obtained.

Polyhydroxyalkanoates (PHA) synthesizing capacity of *Pseudomonas putida* was investigated by genome-scale metabolic model of this microorganism and survival under anaerobic stress was achieved by introducing the *ackA* gene from *Pseudomonas aeruginosa* and *Escherichia coli* (Sohn et al., 2010).

Cai et al. (2011) reported the draft genome of the moderately halophilic bacterium *Halomonas* sp. TD01. In this study, several genes relevant to PHA and osmolytes biosynthesis were analyzed providing invaluable clues for understanding of the evolution and genes transfer, the strategic guidance of the genetic engineering of halophilic *Halomonas* sp. TD01 for co-production of PHA and ectoine.

The analysis of the genes required for the synthesis of the EPS mauran by *H. maura* strain S-30 was performed to identify gene cluster in this strain. Three conserved genes, *epsA*, *epsB*, and *eps*C, also a wzx homolog, *epsJ*, which indicates that mauran is formed by a Wzy-dependent system, were found. It was also reported that eps gene cluster reaches maximum activity during stationary phase, in the presence of high salt concentrations (5% w/v), which was investigated by transcriptional expression assays using a derivate of *H. maura* S-30, which carries an *epsA*: *lacZ* transcriptional fusion (Arco et al., 2005).

Generation of monomers including propanediols, butanediols, diamines, and terpenoids by microorganism through easier biosynthetic pathways was also reported (Lee et al., 2011, 2012; Curran and Alper, 2012). Additionally, production of monomers has been improved using systems biology approaches (Yim et al., 2011; Ng et al., 2012).

Genome-scale metabolic model-guided metabolic engineering approach has been performed and successfully used to improve production yields of various biopolymers and their precursors in synthetic material industry. The microbial production of monomers such as butanediols that are important raw materials in this industry are enhanced by genome-scale metabolic model strategies (Xu et al., 2013). For instance, Ng et al. (2012) have designed and constructed *S. cerevisiae* strains with improved production of 2,3-butanediol with gene deletion strategy, in which disruption of alcohol dehydrogenase (ADH) pathway is required, by performing *in silico* genome-scale metabolic analysis. Yim et al. (2011) have used biopathway prediction algorithm to elucidate possible pathways for 1,4-butanediol (BDO) biosynthesis. Strain development was performed by engineering the *E. coli* host to enhance anaerobic operation of the oxidative tricarboxylic acid cycle and drive the BDO pathway. The engineered strain was able to produce BDO from glucose, xylose, sucrose and biomass-derived mixed sugar streams. Furthermore, the productions of some important carboxylic acid monomers, used as raw materials in synthetic material industry, such as formic acid, malic acid, and succinic acid were improved in engineered *S. cerevisiae* or *E. coli* via genome-scale metabolic models-guided metabolic engineering strategies (Lee et al., 2005b; Wang et al., 2006; Moon et al., 2008; Kennedy et al., 2009). These successfully implemented studies will be helpful to improve microbial EPS production and to design industrial strategies.

Systems metabolic engineering of *E. coli* or *Corynebacterium glutamicum* as efficient cell factories has resulted in overproduction of 1,5-diaminopentane as building block for novel biopolymers (Kind and Wittmann, 2011). The importance of the Entner–Doudoroff pathway in PHB production was predicted by stoichiometric flux analysis of recombinant *E. coli* metabolic model and confirmed experimentally (Hong et al., 2003). The dynamics of PHA copolymer structure and properties were identified by mathematical models during its *in vivo* accumulation (Aldor and Keasling, 2003). Besides, an optimal carbon source switching strategy for the production of block copolymers was described by a population balance model in *Ralstonia eutropha* system (Mantzaris et al., 2001).

Previously genome-scale metabolic model reconstructions of biopolymer producer strain such as *Pseduomonas putida* (Nogales et al., 2008; Puchalka et al., 2008) and *Pseudomonas aeruginosa* (Oberhardt et al., 2008) have been published, as they could be used to elucidate biopolymer synthesis mechanism and improvement of production.

Thermophilic microorganism *Brevibacillus thermoruber* 423 is able to produce high levels of EPS (Yasar Yildiz et al., 2015). Recently, draft genome sequence and whole-genome analysis of this bacterium have been reported. Whole-genome analysis of this bacterium was performed by a systems-based approach to understand the biological mechanisms and whole-genome organization of thermophilic EPS producers. Therefore, strategies for the genetic and metabolic optimization of EPS production could be developed. Genome annotation was used to detect essential genes associated with EPS biosynthesis and a hypothetical mechanism for EPS biosynthesis was generated considering the experimental evidences. The genome sequence of *B. thermoruber* strain 423 is being used to reconstruct a genome-scale metabolic model to develop metabolic engineering strategies since the metabolic model will be used to optimize medium compositions, to modify the microorganism genetically, to improve production yields, and to modify EPS monomer composition (Yasar Yildiz et al., 2014).

Nadkarni et al. (2014) performed comparative genome analysis of *Lactobacillus rhamnosus* clinical isolates to identify EPS cluster. In this study, transcriptional orientation of the eps cluster genes, the presence of two genes homologous to priming glycosytransferases, the absence of *rmlACBD* genes involved in the dTDP-rhamnose biosynthetic pathway, and the presence of a family 2 GTF in the eps cluster of both clinical isolates of *L. rhamnosus*, is predicted to alter EPS composition and could influence pathogenicity.

The first complete genome sequence of Russia origin *Bifidobacterium longum* subsp. longum strain GT15, comparative genome analysis, identification, and characterization of regulatory genes, *in silico* analysis of all the most significant probiotic genes and considered genes have been reported. The genomic analysis for polysaccharides was also performed, and it was observed that most of the genes in the carbohydrate metabolism category were involved in the utilization of oligo-polysaccharides. The genome also contains genes predicted to encode proteins involved in the production of capsular EPS, which are most likely involved in bacteria–host interactions (Zakharevich et al., 2015).

Genome sequence of moderately halophilic and EPS-producing *Salipiger mucosus* DSM 16094T and the presence of a high number of genes associated with biosynthesis of EPSs have been reported. Genes associated with the synthesis of polyhydroxyalkanoates have been also found (Riedel et al., 2014).

*Acidithiobacillus ferrooxidans* was the first biomining microorganism whose genome was sequenced and the genes involved in the biosynthesis EPSs precursors have been studied (Valdés et al., 2008). The cluster of five genes proposed to be involved in the biosynthesis of EPSs precursors via the Leloir pathway have been also identified previously (Barreto et al., 2005).

A curdlan producer *Agrobacterium* sp. ATCC 31749’s genome was sequenced and the curdlan biosynthesis operon (crdASC) was identified (Ruffing et al., 2011). Moreover, transcriptome analysis of this microorganism has been performed to understand the regulation of EPS biosynthesis (Ruffing and Chen, 2012). In this study, transcriptome profiling was used to identify genes that expressed during curdlan biosynthesis and carry out targeted gene knockouts to investigate their roles in the transcriptional regulation of curdlan production. The analysis showed that curdlan synthesis operon was upregulated by up to 100-fold upon nitrogen depletion. Moreover, novel regulation mechanisms including RpoN-independent NtrC regulation and intracellular pH regulation by acidocalcisomes were identified.

Engineering studies for LAB, particularly members of the genera *Lactococcus* and *Lactobacillus*, have been performed for the production of platform chemicals, such as lactate l- and d-stereoisomers,1,3-propanediol, and 2,3-butanediol, food flavors and sweeteners, vitamins, and complex polysaccharides (Gaspar et al., 2013).

Exopolysaccharide biosynthetic pathways in LAB have been engineered to have challenges greater than manipulating specific steps in primary metabolism (Patel et al., 2011). Since EPSs enhance potential health benefits of fermented food products, many metabolic engineering approaches are employed to improve productivity and structure of EPS (Gaspar et al., 2013).

Omics studies can be used to improve the understanding of metabolism in food industry microorganisms. The metagenomic studies for fermented food were performed to analyze the metabolic potentials of LAB bacteria, which is very important in industrial fermentations. Jung et al. (2011) performed metagenomic studies changes in bacterial populations, metabolic potential, and the overall genetic features of the microbial community during a 29-day fermentation process of the traditional Korean food kimchi. The transcriptome response has been analyzed in yogurt fermentation (Sieuwerts et al., 2010) and in milk (Goh et al., 2011).

The first genome-scale model for *L. lactis* (Oliveira et al., 2005), and since whole-genome metabolic reconstructions for *Lb. plantarum* (Teusink et al., 2005) and *S. thermophilus* (Pastink et al., 2009) have been reported. Functional genomics and other studies have performed to investigate genomic diversity in LAB and the findings highlighted the variety of carbon substrates potentially used by LAB including simple sugars, complex carbohydrates such as xylan, starch, and fructans, α-galactosides (e.g., raffinose and stachyose), pentoses (D arabinose and D-xylose), and the cheap C3 carbon source glycerol (Teusink et al., 2009; Siezen and van Hylckama Vlieg, 2011).

Complete genomic sequence of *Lb. bulgaricus* 2038 has been reported and genomic analysis of EPS biosynthesis has been performed. Two neighboring *eps* clusters with significant differences were identified when compared with genome sequence of *Lb. bulgaricus* species by comparative genomic analysis (Hao et al., 2011).

Genomic studies microbial EPSs producer of deep-sea bacteria such as *Zunongwangia profunda* SM-A87, *Pseudoalteromonas* sp. SM9913, *Pseudoalteromonas haloplanktis* TAC125 (Qin et al., 2011), have been also performed and analyzed for EPS gene clusters (Finore et al., 2014). In addition, genome sequence of several deep-sea isolates such as *Idiomarina loihiensis* (Hou et al., 2004) and *Alteromonas macleodii* (Ivars-Martinez et al., 2008) demonstrated the EPS biosynthesis genes (Finore et al., 2014).

Such omic studies and works from systems biology perspective will play an important role both scientifically and economically, since there is a great need for developing efficient methodologies for enhanced EPS biosynthesis. More information on the genome of the microorganism will enable to develop strategies to successfully enhance production rate and also to engineer EPSs properties by modifying composition and chain length. Systems-based modeling approach constitutes an important step toward understanding the interplay between metabolism and EPS biosynthesis.

Systems biology approaches and metabolic reconstruction studies of microbial EPS have been also reported for two important EPSs: xanthan and levan. These studies and their findings were given in detail in the following sections. Besides, these two important EPSs researches on pullulan and dextran were also discussed and the general properties and applications for all these important EPSs were summarized in Table 1.

**Table 1:** **General description of significantly important microbial EPSs**.

Microbial EPS	Monomeric units	Microorganism	Industrial applications	Omic studies	Metabolic model
Xanthan	Glucose, mannose and glucuronate	*Xanthamonas* sp.	Thickening, stabilizing agent, food additive, etc.	Genome sequence, Proteomics	Available (Schatschneider et al., 2013)
Levan	Fructose	*Halomonas smyrnensis* AAD6^T^, *Zymomonas mobilis*, *Bacillus subtilis (natto)*	Emulsifier, stabilizer and thickener, encapsulating agent, food and feed additive, osmoregulator, and cryoprotector, etc.	Genome sequence	Available (Ates et al., 2013; Diken et al., 2015)
Pullulan	Glucose	*Aureobasidium pullulans*	Thickening, stabilizing, texturizing, gelling agents, etc.	Genome sequence, proteomics, genome shuffling	Not available
Dextran	Glucose	*Leuconostoc* spp. and *Streptococcus* spp.	Blood plasma extender and chromatography media	Genome sequence	Not available

## Xanthan

The commercially most important microbial EPS is known as Xanthan which is produced by the plant-pathogen-proteobacterium *Xanthomonas campestris* pv. campestris (Xcc) (Vorhölter et al., 2008; Frese et al., 2014). It is a heteropolysaccharide composed of repetitive pentasaccharide units consisting of monomeric units of two glucose, two mannose, and one GlcA residues with with a backbone chain consisting of (1-4)-β-d-glucan cellulose (Khan et al., 2007). Due to its superior properties and rheological characteristics, xanthan has found a wide range of applications as a thickening or stabilizing agent in food, cosmetics and oil drilling industries (Schatschneider et. al, 2013; Chivero et al., 2015). It has been described as a “benchmark” product based on its significance in food and non-food applications which include dairy products, drinks, confectionary, dressing, bakery products, syrups, and pet foods, as well as the oil, pharmaceutical, cosmetic, paper, paint, and textile industries (Patel and Prajapati, 2013; Cho and Yoo, 2015). Xanthan was also employed in non-food applications such stabilizing cattle feed supplements, calf milk substitutes, agricultural herbicides, fungicides, pesticides, and fertilizers, and to impart thixotropy into toothpaste preparations (Morris, 2006). Considering the commercial importance of this microbial EPS, omics studies and metabolic model reconstructions were performed to clarify xanthan biosynthesis mechanism.

The genomes of five *Xanthomonas* strains *X. campestris* pv. campestris strains ATCC 33913 (da Silva et al., 2002) and 8004 (Qian et al., 2005), *X. campestris* pv.vesicatoria strain 85-10 (Thieme et al., 2005), *X. oryzae* pv. oryzae strains KACC10331 (Lee et al., 2005a,b) and MAFF 311018 (Ochiai et al., 2005), *X. axonopodis* pv. citri strain 306 (da Silva et al., 2002) have been sequenced. The complete genome sequence of the xanthan producer strain *Xanthomonas campestris* pv. campestris strain B100 and its use for mechanistic model for biosynthesis of xanthan have been also reported (Vorhölter et al., 2008). In this study, the gene products and metabolic pathways for xanthan polymerization were investigated in detail. The gene products of *gumJ, gumC, gumD*, and *gumE* were analyzed to establish detailed functions in a xanthan polymerization. Moreover, the mechanistic model for the biosynthesis of xanthan was established.

*In vivo* proteome analysis *X. campestris* pv. *campestris* has been performed to investigate protein expression of the microorganism during host–plant interaction. Peptide mass fingerprinting or *de novo* sequencing methods were utilized for identification of the functions of proteins. This approach will be used to determine the roles of proteins in pathogenicity mechanismsand also xanthan biosynthesis (Andrade et al., 2008).

Schatschneider et al. (2013) has reported the first large-scale metabolic model for *Xanthomonas campestris* (Xcc), which was reconstructed from genome data of, manually curated and further expanded in size. The impact of xanthan production was studied *in vivo* and *in silico* and compared with *gumD* mutant strain. This verified metabolic model is also the first model focusing on bacterial EPS synthesis and it can be used for detailed systems biology analyses and synthetic biology reengineering of Xcc. Moreover, draft genome of *X. campestris* B-1459, which was used in pioneering studies of xanthan biotechnology, and it can be used to analyze the genetic basis of xanthan biosynthesis has been reported recently (Wibberg et al., 2015).

## Levan

Levan is a naturally occurring polymer that is composed of β-d-fructofuranose with β(2-6) linkages between fructose rings. It is synthesized by the action of a secreted levansucrase (EC 2.4.1.10) that directly converts sucrose into the polymer (Han and Clarke, 1990). As a homopolysaccharide with many distinguished properties such as high solubility in oil and water, strong adhesivity, good biocompatibility, and film-forming ability, it has great potential as a novel functional biopolymer in foods, feeds, cosmetics, pharmaceutical, and chemical industries (Kang et al., 2009; Kazak Sarilmiser et al., 2015). In fact, a recent literature analysis on microbial EPSs attributed levan together with xanthan, curdlan, and pullulan as the most promising polysaccharides for various industrial sectors (Donot et al., 2012).

Due to its exceptionally high production costs, levan could never find its proper place in the polymer market, and therefore, high-level levan producing microbial systems gain escalating industrial importance. Levan is produced as an EPS from sucrose-based substrates by a variety of microorganisms, including the halophilic bacterium *Halomonas smyrnensis* AAD6^T^, which has been reported as the first levan producer extremophile (Poli et al., 2009).

The gram-negative halophilic bacterium *H. smyrnensis* AAD6^T^, which was isolated from Çamaltı Saltern Area in Turkey (Poli et al., 2009, 2013), was found to excrete high levels of levan (Poli et al., 2009). With this microbial system, productivity levels were further improved by use of cheap sucrose substitutes such as molasses (Kucukasik, 2010; Kucukasik et al., 2011) as well as other cheap biomass resources (Toksoy Oner, 2013) as fermentation substrate. Further research on the potential uses of levan produced by *H. smyrnensis* AAD6^T^ as a bioflocculating agent (Sam et al., 2011), its nanostructured thin films (Sima et al., 2011, 2012), its suitability for peptide-based drug nanocarrier systems (Sezer et al., 2011), and its adhesive mulitilayer films (Costa et al., 2013) have been reported.

Increasing significance of levan in industrial and medical biotechnology calls for the elucidation of the interrelations between metabolic pathways and levan biosynthesis mechanism in order to control and hence enhance its microbial productivity. However, there is very limited information about the mechanisms involved in the biosynthesis of levan from extremophiles (Nicolaus et al., 2010) and no report about a systematic approach to analyze levan production by *H. smyrnensis* AAD6^T^. Considering this fact, systems-based approaches were applied to improve the levan production capacity of *H. smyrnensis* AAD6^T^ cultures (Ates et al., 2013).

The genome sequence forms the basis for metabolic model reconstruction; however, there was a lack for genome information of *H. smyrnensis* AAD6^T^. Only recently, its draft genome sequence has been announced. *De novo* assembly of the whole sequencing reads were carried out in this study. Consequently, several genes related to EPS biosynthesis, including the genes for levansucrase and *ExoD* were revealed by genome analysis (Sogutcu et al., 2012). Due to the absence of genomic information, first, comprehensive metabolic model of a taxonomically close halophilic bacterium, namely, *C. salexigens* DSM3043 have been reconstructed (Ates et al., 2011). Then, in order to investigate levan biosynthesis by a metabolic systems analysis approach, the genome-scale metabolic network of *C. salexigens* was recruited and adopted to *H. smyrnensis* AAD6^T^ via integration of the available biochemical, physiological, and phenotypic features of *H. smyrnensis* AAD6^T^. The *in silico* metabolic model was verified with dynamic experimental data on different medium compositions and was then systematically analyzed to identify critical network elements (i.e., enzymes, biochemical transformations, and metabolites) related to levan biosynthesis mechanism. The findings manifested mannitol as a significant metabolite for levan biosynthesis, which was further verified experimentally. In the previous study, 1.844 g/L levan yield from the stationary phase bioreactor cultures using a defined media containing sucrose as sole carbon source with almost fourfold increase of levan production was reported (Ates et al., 2013).

Recently, Diken et al. (2015) performed the whole-genome analysis of *H. smyrnensis* AAD6^T^ to investigate biological mechanisms, and furthermore, the genome-scale metabolic model *i*KYA1142, which included 980 metabolites and 1142 metabolic reactions, was reconstructed. The genomic analysis figured out the biotechnological potential of this microorganism as a result of its capacity to produce levan, Pel exopolysaccharide, polyhydroxyalkanoates (PHA), and osmoprotectants. Genes related to EPS biosynthesis and intracellular PHA biosynthesis were detected. *Hs_SacB* gene encoding the extracellular levansucrase enzyme (EC 2.4.1.10), Pel polysaccharide gene cluster (*PelA, PelB, PelC, PelD, PelE, PelF*, and *PelG*), Alginate lyase precursor (EC 4.2.2.3), and “Alginate biosynthesis protein Alg8” genes were predicted. The genome information and metabolic model will have a significant role on levan biosynthesis since they will be utilized to improve levan production by metabolic engineering strategies and medium optimization.

## Pullulan

A fungal glucan “pullulan” that is a linear homopolysaccharide composed of maltotriose reduplicative units connected by α-1,4-linkages is produced by *Aureobasidium pullulans* (Singh et al., 2008; Sheng et al., 2014; Özcan et al., 2014). Although pullulan production studies mostly focused on *A. pullulans*, other producer strains such as *Remella mesenterica*, *Cryphonectria parasitica*, and *Teloschistes flavicans* were also reported (Cheng et al., 2011). Biosynthesis of pullulan was occurred intracellularly at the cell wall or cell membrane and microorganisms secreted this EPS out to the cell surface to form a loose and slimy layer (Ma et al., 2015). Its solubility in water is excellent as a result of the linkage pattern. Moreoever, it has outstanding chemical and physical properties, such as low viscosity, non-toxicity, slow digestibility, high plasticity, and excellent film-forming (Cheng et al., 2011; Sheng et al., 2014).

The major market for pullulan is food industry and also its potential applications in pharmaceutical, agricultural, chemical, cosmetic, biomedical, and environmental remediation areas have been reported (Özcan et al., 2014; Ma et al., 2015). Due to its superior properties, pullulan can be used as non-polluting wrapping material for food supplements, oxygen impermeable, edible, and biodegradable and highly water soluble films, denture adhesives, emulsifiers and stabilizers for various food products, binder, lubricant, gelling agent, oral care products, and blood plasma substitute. Since it is non-immunogenic, non-toxic, non-carcinogenic, and non-mutagenic, pullulan can find novel application areas such as gene therapy, targeting drugs, and gene delivery (Cheng et al., 2011; Singh et al., 2015). Pullulan has been commercialized in various countries, used as a safe food ingredient and pharmaceutical bulking agent and in Japan.

The molecular biosynthesis of pullulan is a complex metabolic process therefore the molecular basis of this process is not clearly identified. The three significant enzymes for pullulan biosynthesis are α-phosphoglucose mutase (PGM, EC 5.4.2.2), UDP-glucose pyrophosphorylase (UGP, EC 2.7.7.9), and glucosyltransferase (FKS, EC 2.4.1.34). Nitrogen is the major component for the cultivation of *A. pullulans* in pullulan fermentation (Wang et al., 2015). Wang et al. (2015) analyzed gene expression of the key enzymes (PGM, UGP, FKS) to improve pullulan production. They have reported the improved pullulan production correlated to the high activities of PGM and FKS, increased the activities of α-phosphoglucose mutase and glucosyltransferase, and upregulated the transcriptional levels of pgm1 and fks genes by nitrogen limitation.

Kang et al. (2011) studied genome shuffling *A. pullulans* N3.387 by ethyl methane sulfonaten (EMS) and ultraviolet (UV) mutagenesis to improve pullulan biosynthesis and developed a mutant that could produce more pullulan than wild type strain.

Sheng et al. (2014) performed proteomic studies of pullulan production in *A. pullulans* to understand the effect of different concentrations of (NH_4_)_2_SO_4_ which would be useful to optimize industrial pullulan production. The proteomic studies demonstrated the expression of antioxidant related enzymes and energy-generating enzymes and the depression of the enzymes concerning amino acid biosynthesis, glycogen biosynthesis, glycolysis, protein transport, and transcriptional regulation under nitrogen limitation, resulted in conversion of metabolic flux from the glycolysis pathway to the pullulan biosynthesis pathway.

Draft genome of *Aureobasidium pullulans* AY4 was determined and genome analysis revealed the presences of genes coding for commercially important enzymes such as pullulanases, dextranases, amylases, and cellulases (Chan et al., 2012). Gostin et al. (2014) performed *de novo* genome sequencing and genome analysis of the four varieties of *A. pullulans* to investigate the genomic basis of pullulan biosynthesis potential. Single-copy genes for phosphoglucose mutase and uridine diphosphoglucose pyrophosphorylase, which are the key enzymes for converting glucose units into pullulan, were present in four varieties of *A. pullulans*. Genomic analysis also revealed that these microorganisms included all of the putative enzymes that were proposed to be involved in pullulan biosynthesis (Gostin et al., 2014). The genome sequences of *A. pullulans* species will be facilitated to clarify biosynthesis mechanism of pullulan and improve its production.

## Dextran

Dextran, which is a homopolysaccharides composed of α-1,6 glycosidic linkages, is produced by *L. mesenteroides* hydrolases in the precense of sucrose. Various strains of bacteria such as *Streptococcus* and *Acetobacter* have been also found to produce dextran bacteria (e.g., *Leuconostoc* and *Streptococcus*), through the use of specific enzymes like glucansucrases (Patel et al., 2011; Casettari et al., 2015). Since dextran has a flexible structure as a result of free rotation of glycosidic bond and it is highly soluble in water, biocompatible, and biodegradable, it becomes a functional hydrocolloid (Ahmad et al., 2015). It has commercial applications in food, pharmaceutical and chemical industries as adjuvant, emulsifier, thickener, carrier, and stabilizer. Moreoever, dextran is used as therapeutic agent to restore blood volume, for the matrix preparation of chromatography columns, for synthesizing dextran sulfate for blood coagulation prevention and blood flow facilitation, as osmotic agents, lubricant in eye drops and for increase of blood sugar levels, iron carrier, anticoagulant for pharmacy (Patel et al., 2011; Han et al., 2014).

Dextrans have also found use in different areas such as veterinary medicines, biosensor material, food syrup stabilizers, dough improvers, metal-plating processes, enhanced oil recovery, stabilizing coating for protecting metal nanoparticles against oxidation, and coating on biomaterials to prevent undesirable protein absorption. Dextran and its derivatives such as cyclodextran are utilized in the pharmaceutical industry like cariostatic, anti-HIV, and anti-ulcer agent (Ahmad et al., 2015; Casettari et al., 2015).

Various genome sequence and genome analysis studies were carried out with dextran producer strains. For instance, the genome of LAB *Leuconostoc gasicomitatum* was sequenced and genome analysis was performed to understand the growth and spoilage potentials. The genomic analysis revealed genes for two dextransucrases catalyzing the formation of dextran from sucrose: *epsA* (LEGAS_0699) is part of a large EPS cluster, while *dsrA* (LEGAS_1012) is located as a single gene in the chromosome (Johansson et al., 2011). Saulnier et al. (2011) performed the metabolic pathway reconstruction, genome profiling, genomic, and transcriptomic comparisons of the *Lactobacillus reuteri* strains to define functional probiotic features. Genome-wide comparison resulted in dextranase gene that predicted to encode the synthesis of EPS. The first genome sequence of *Weissella* species has been announced for *Weissella confusa* (formerly *Lactobacillus confusus*) LBAE C39-2, which was also found promising for *in situ* production of dextran in sourdoughs (Amari et al., 2012). A draft genome sequence of dextran producer *Leuconostoc lactis* EFEL005 has been announced and genomic analysis was performed to understand its probiotic properties as a starter for fermented foods (Moon et al., 2015). The increased number of genome sequences will accelerate systems-based works for dextran biosynthesis mechanism.

## Conclusion and Recommendations

In microbial EPS production, a better understanding of biosynthesis mechanism is a significant issue for optimization of production yields, improvement of product quality and properties, and also for the design of novel strains. As most of the novel bacterial EPS with unique properties have expensive production costs and economic hurdles need to be overcome, this valuable information about biosynthesis is also be important to lower these charges.

More information on the genome of the EPS producer microorganisms will enable to develop additional strategies to successfully enhance EPSs production rate and also to engineer their properties by modifying composition and chain length. Since genome-scale reconstruction includes every reaction of target organism through integrating genome annotation and biochemical information, a systems-based metabolic modeling approach constitutes an important step toward understanding the interplay between metabolism and EPSs biosynthesis. Since microbial biopolymer biosynthesis is a result of a complex system of many metabolic processes, systems-based approaches are needed to control and optimize production in order to improve the formerly reported yields.

Furthermore, the genome-scale metabolic model based on genome sequence will have the capacity to consider gene expression, metabolomics, and proteomics data to get accurate prediction at different environmental conditions.

## Conflict of Interest Statement

The author declares that the research was conducted in the absence of any commercial or financial relationships that could be construed as a potential conflict of interest.
